# Characteristics and stability of sensorimotor activity driven by isolated-muscle group activation in a human with tetraplegia

**DOI:** 10.1038/s41598-022-13436-2

**Published:** 2022-06-20

**Authors:** Robert W. Nickl, Manuel A. Anaya, Tessy M. Thomas, Matthew S. Fifer, Daniel N. Candrea, David P. McMullen, Margaret C. Thompson, Luke E. Osborn, William S. Anderson, Brock A. Wester, Francesco V. Tenore, Nathan E. Crone, Gabriela L. Cantarero, Pablo A. Celnik

**Affiliations:** 1 Department of Physical Medicine and Rehabilitation, Johns Hopkins School of Medicine, Baltimore, MD USA; 2 Department of Biomedical Engineering, Johns Hopkins School of Medicine, Baltimore, MD USA; 3 Department of Neurology, Johns Hopkins School of Medicine, Baltimore, MD USA; 4 Department of Neurosurgery, Johns Hopkins School of Medicine, Baltimore, MD USA; 5 Research and Exploratory Development Department, Johns Hopkins Applied Physics Laboratory, Laurel, MD USA; 6 National Institute of Mental Health, National Institutes of Health, Bethesda, MD USA; 7 Department of Neuroscience, Johns Hopkins School of Medicine, Baltimore, MD USA

**Keywords:** Brain-machine interface, Neurophysiology

## Abstract

Understanding the cortical representations of movements and their stability can shed light on improved brain-machine interface (BMI) approaches to decode these representations without frequent recalibration. Here, we characterize the spatial organization (somatotopy) and stability of the bilateral sensorimotor map of forearm muscles in an incomplete-high spinal-cord injury study participant implanted bilaterally in the primary motor and sensory cortices with Utah microelectrode arrays (MEAs). We built representation maps by recording bilateral multiunit activity (MUA) and surface electromyography (EMG) as the participant executed voluntary contractions of the extensor carpi radialis (ECR), and attempted motions in the flexor carpi radialis (FCR), which was paralytic. To assess stability, we repeatedly mapped and compared left- and right-wrist-extensor-related activity throughout several sessions, comparing somatotopy of active electrodes, as well as neural signals both at the within-electrode (multiunit) and cross-electrode (network) levels. Wrist motions showed significant activation in motor and sensory cortical electrodes. Within electrodes, firing strength stability diminished as the time increased between consecutive measurements (hours within a session, or days across sessions), with higher stability observed in sensory cortex than in motor, and in the contralateral hemisphere than in the ipsilateral. However, we observed no differences at network level, and no evidence of decoding instabilities for wrist EMG, either across timespans of hours or days, or across recording area. While map stability differs between brain area and hemisphere at multiunit/electrode level, these differences are nullified at ensemble level.

## Introduction

Brain—machine interfaces (BMIs) to restore motor function rely on decoders to infer intended movements from sensorimotor neural activity. Decoder efficacy depends both on the relevance of neural regions sampled—how reliably they map to executed (or attempted) movements—and the stability of the regions—how consistent their activity patterns are over time.

Many efforts have been made to topographically map the body onto the primary motor (M1) and sensory cortices (S1)^1–4^. Historically, M1 stimulation showed that individual body parts are represented with a partially-fractionated somatotopy; namely with the face, arm, and legs seated in largely distinct areas, and individual muscles within an effector represented in a more mixed and overlapping fashion^5,6^. A recent study using microelectrode arrays (MEAs) implanted in a human BMI participant, however, provided evidence of intermixed whole-body tuning within the hand knob area^7^, raising questions about M1 organization. The primary somatosensory cortex (S1)^3^, by contrast, exhibits particularly clear somatotopy for cutaneous touch, with proprioception having relatively greater overlap between neighboring regions^8,9^.

Although much work has focused on characterizing the layout of the sensorimotor map, we know less about its stability. One can examine stability in the neural representation of a given movement at multiple levels: as consistencies in the location of active neurons (somatotopy), in the firing patterns at individual recording sites (within-channel level), and in the activation pattern across all electrodes (network or ensemble level). Studies of neural stability at within-channel and cross-channel (ensemble) levels have been limited to motor areas and focused mostly on non-human primates (NHPs) without spinal injuries^10–16^. At the within-electrode level, there is conflicting evidence about whether firing rates or tuning patterns stay consistent for several days^17–20^ or whether they are more typically limited to more modest periods of several minutes to a few hours^20–22^. Studies at the ensemble or network level have shown that the representation between network-wide activity and reaching movements may be stable through multiple weeks in NHPs^23,24^, and even a few years in the context of an ecologically important task like targeted reaching^18^. Unfortunately, replications of this approach in humans are limited because of a comparative shortage of studies and the lack of intact, consistently replicable movements to analyze in BMI patients.

Questions of stability are practically relevant. BMIs trained in humans have largely failed to replicate performance in NHPs, often needing to be retrained multiple times throughout sessions^23,25–33^. Understanding how stable these maps are and whether their underlying neural activity patterns persist over time may be key to developing longer-lasting decoders. As human BMI usage grows, further studies of body map organization and stability are needed, both to understand how implant location affects signal consistency and quality, and to address recent challenges to the principle of somatotopic organization from MEAs implanted in humans.

Here, we studied representation of wrist muscle contractions and its stability in a tetraplegic human (C5/6 incomplete, ASIA B), the first person to be implanted bilaterally with MEAs in the traditional hand area representation of precentral (M1) and postcentral (S1 Area 1) gyri^34–36﻿^. First, we estimated the sensorimotor map associated with EMG-controlled muscle contractions (or attempted motions) in the forearm above and below injury level. Then we characterized stability of neural activity throughout the map over varying time periods for the wrist extensor muscles (extensor carpi radialis: ECR) of both left and right arms. We investigated stability from multiple perspectives, namely spatial patterning (somatotopy), electrode-level signaling, and ensemble-level signaling across brain hemispheres and sensorimotor cortices.

## Results

**Figure 1:**
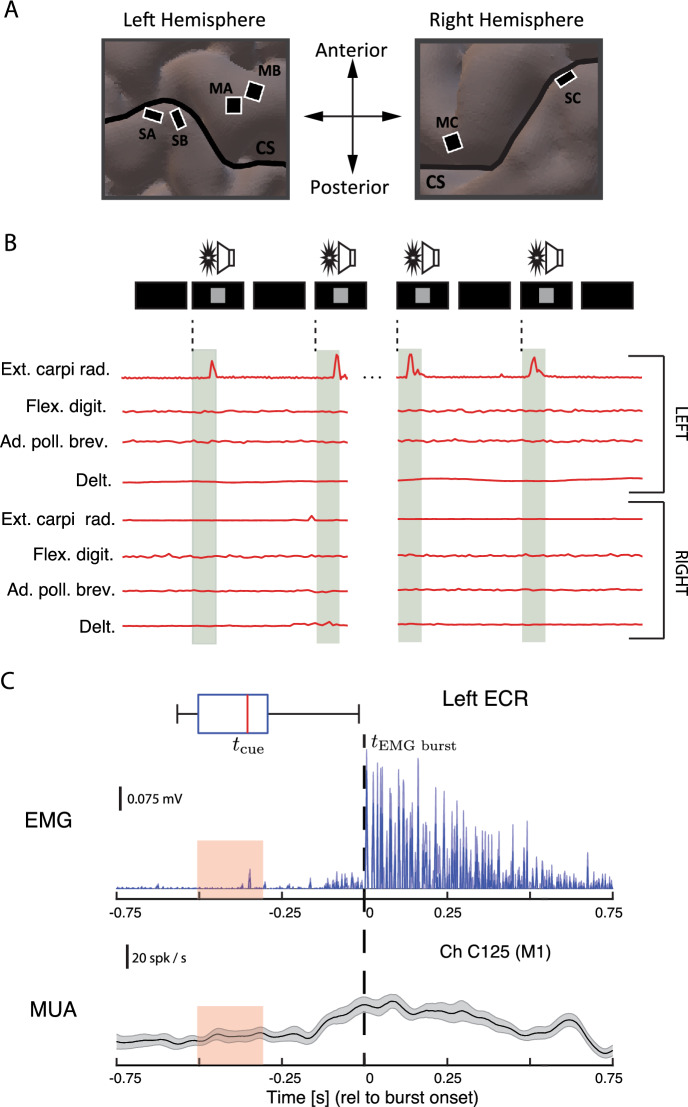
Cortical recording sites and experimental methods. (**A**) Sites of the six bilaterally-implanted microelectrode arrays (MEAs), overlaid on MRI reconstruction of participant’s brain (CS: central sulcus). Arrays are labeled by anatomical target and pedestal (e.g. MA: primary motor cortex, pedestal A; SB: primary sensory cortex, pedestal B). (**B**) Experimental paradigm. Isolated muscle contractions (or contraction attempts) were cued by metronome ticks (click and pixel flash) at 4-s intervals. Electromyography (EMG) traces are from 4 example trials (repetitions), where the participant executed left extensor carpi radialis (ECR) contractions without co-contracting neighboring or opposite-limb muscles. (**C**) Temporal referencing and synchronization of neuromuscular and cortical data for a representative EMG-producing muscle (the left extensor carpi radialis: ECR). The upper plot shows event-referenced raw EMG in mV (reference line). Box plot shows movement cue time distributions relative to burst onset; pink regions are the interquartile range (IQR) of this distribution. The lower plot shows the peri-event time histograms (PETH) of multiunit activity (MUA) from an example motor channel on the contralateral motor array, in spikes/sec. Signals were referenced to EMG burst onset (dashed line; see Supplementary Information), and are trial averaged. Shaded regions show bootstrapped 95%–confidence intervals.

**Figure 2:**
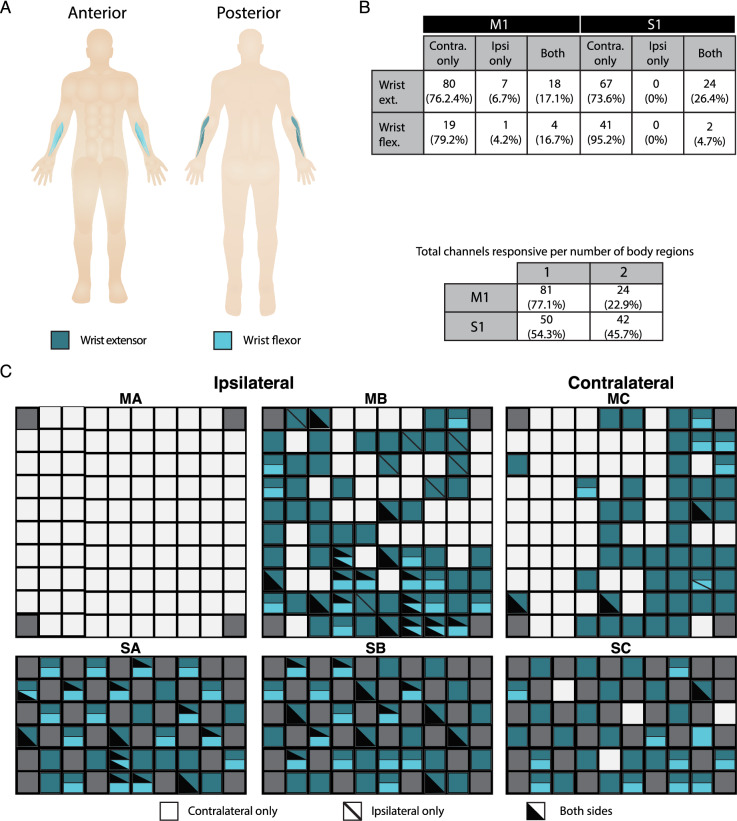
Regional body map for wrists, showing activity patterns across individual muscles. **A**: Overview of assessed muscles, color-coded by group. Targeted groups were: wrist extensors–-extensor carpi radialis; wrist flexors: flexor carpi radialis. **B**: Summary of channel-level representation of each muscle by brain area (M1: motor cortex arrays; S1: sensory cortex arrays) and laterality (contralateral, ipsilateral, or both). Counts are raw numbers, and percentages are with respect to all channels active for that region within the indicated brain area. **C** Activity maps for right hemisphere (MA: motor array, pedestal A; MB: motor array, pedestal B; SA: sensory array, pedestal A; SB: sensory array, pedestal B) and left hemisphere (MC: motor array, pedestal C; SC: sensory array, pedestal C). Channels with significant MUA responses to contractions in a body region are colored as in panel A. Solid colors indicate activity for only the contralateral side of the body (right side for MA/SA/MB/SB, left side for MC/SC); diagonal lines denote activity for the ipsilateral side; and black triangle overlays denote that both sides of the body elicited a response.

During each experimental session, we measured neural activity associated with isolated EMG-controlled muscle contractions using a metronome-paced task (Fig. 1; see Materials and Methods for details). Given the location of the electrode implants (in the hand knob of motor cortex and hand representations of sensory cortex: Fig. 1A), we focused on contractions of the ECR, since our participant retained voluntary control of these muscles, and (attempted movements of the flexor carpi radialis (FCR), an antagonist for which voluntary control was lost (Fig. 2). Sessions consisted of blocks where we instructed the participant to contract (or attempt to contract) a specific muscle in isolation to an audiovisual metronome ticking every 4 s (Fig. 1B). To assure that contractions were isolated, we trained the participant’s movements under guidance of physical therapists, and simultaneously monitored electromyograms (EMG) for the instructed muscles and surrounding ones likely to be co-activated. As shown in Fig. 1C, muscle contractions elicited MUA responses, which we quantified in terms of peri-event time histograms (PETH; see Materials and Methods). Subsequent analyses on PETHs were based on windows relative to the burst onset. We labeled as noncompliant any trials where EMG was absent or the participant co-contracted muscles outside of the wrist, and excluded such noncompliant trials from further analysis.

To verify the coverage of our MEAs, we tested contractions of the ECR and FCR of both arms (Fig. 2A,B). Figure 2B tabulates, for each muscle, significantly active channels and their laterality (contralateral, ipsilateral, or bilateral). Overall, 105 channels in M1 and 92 channels in S1 responded to contractions within at least one wrist muscle. Significant activity manifested in all motor and sensory arrays except for MA. Although muscle contractions could be well isolated to one side of the body, channel activity was present in both hemispheres.

Unilateral activity accounted for 82.9% of active M1 and 73.6% active S1 channels for the extensor contractions, and 83.3% of active M1 and 95.2% of active S1 channels for flexor contractions. Critically, in a control experimental session where the participant sat quietly listening to the same metronome, we found no significant channel activations on any array.

To study map stability, we repeated and analyzed the mapping experiment across 11 sessions (see Table S1 in Supplementary Information). Our main focus was the left ECR since the participant could isolate this muscle most consistently; however, for comparison, we also collected and analyzed data from the right ECR. Although data were collected from a 12th session (409 days after array implantation), we excluded them as outliers because fewer than 10 channels yielded significant activations on that date.

We quantified task performance by the number of trials with task errors per session (non-responses, muscle co-contractions, or unclean contractions without clear EMG onset; see Fig. S1). Lack of a clear error pattern across sessions indicated that learning was not a major factor in our task. Moreover, total activated channels did not vary significantly across days (Fig. S2), except in the contralateral (right) sensory cortex for left ECR contractions, where there was a positive correlation with time (Kendall’s tau($\tau$) = 0.40, *p* = 0.01).

We assessed stability at three levels in all compliant ECR contraction trials. First, we examined the consistency and location of active channels across sessions, which we term *longitudinal spatial stability*. Second, within channels, we measured similarities of PETH amplitude (firing strength stability) and PETH shape (*firing dynamic stability*) over adjacent time periods. Finally, we compared activity patterns across populations of channels, based on the similarity of trajectories on a neural manifold, which we call *ensemble stability.* We provide further details in the Materials and Methods, and Supplementary Information sections.

### Spatial stability persists longer in the contralateral hemisphere than the ipsilateral, and in sensory areas than motor

**Figure 3:**
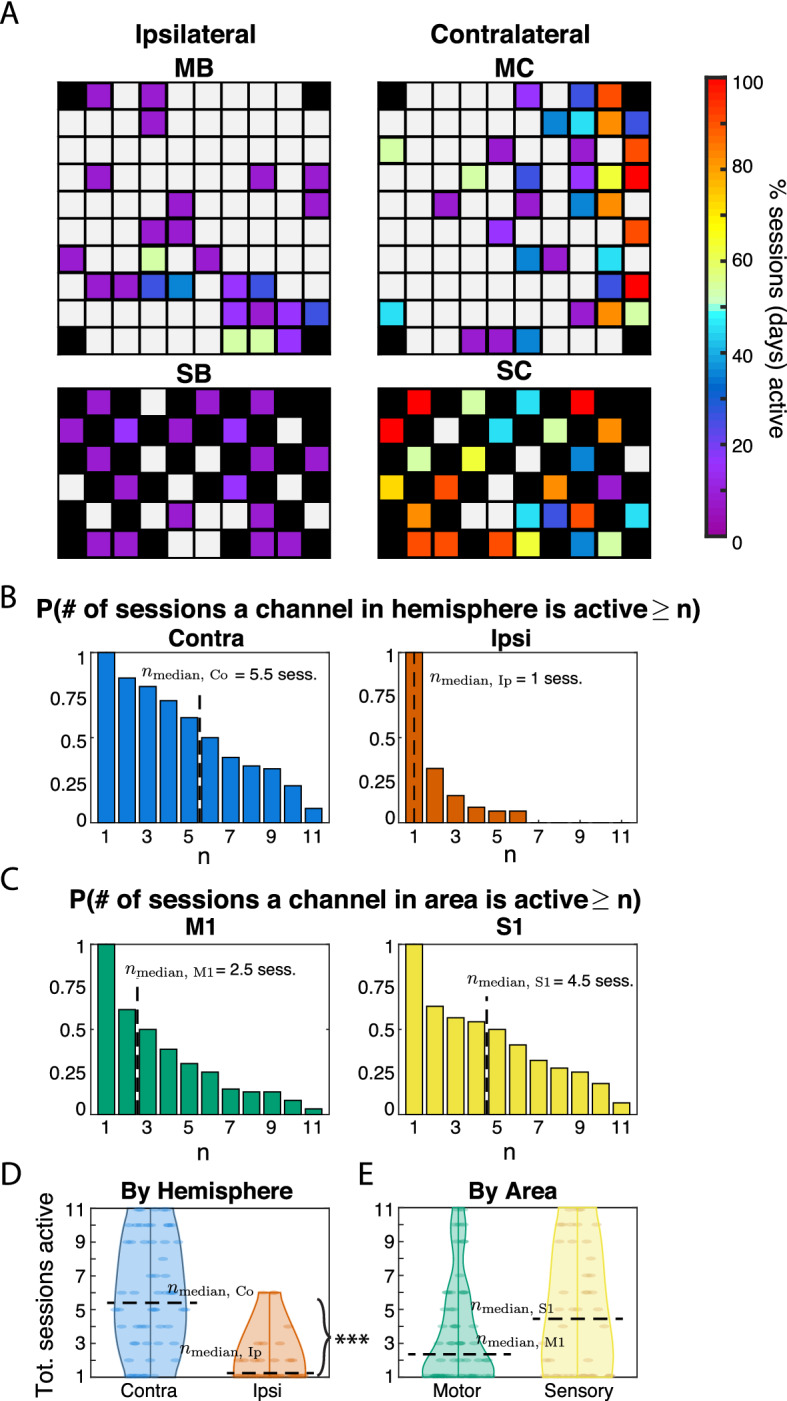
Spatial patterning and longitudinal stability activity from contractions of left wrist extensor (ECR: extensor carpi radialis). Longitudinal stability is considered relative to how often a channel is measured as active relative to the number of experiment sessions (11 total). (**A**) Frequency of activity across sessions for each channel distributed over arrays B and C (top: motor; bottom: sensory). Color code denotes the percentage of sessions (of 11) that a given channel was active, with higher percentages corresponding to greater longitudinal stability. (**B**) Probabilities that any active channel on Pedestals B and C responded for more than n sessions, within contralateral (left panel) and ipsilateral (right) hemispheres. Motor and sensory areas are pooled. Dashed lines mark the median number of sessions responsive among channels within the active footprint of each hemisphere. (**C**) Probabilities that any active channel on Pedestals B and C (mapped in panel A) responds for more than n sessions, measured for motor (left panel) and sensory (right) areas (hemispheres pooled). For example, a channel active for exactly 2 out of 11 possible sessions would be counted in the bars for both n = 1 and n = 2. Dashed lines mark the median number of sessions that a given channel in the active footprint of each area responds. (**D**) Distributions of the number of total sessions a channel responds to attempted left wrist extensions, by hemisphere. The median number of sessions a channel was observed responsive was greater within the contralateral than the ipsilateral hemisphere (n_Median, Co_–n_Median, Ip_ = 5 sessions; *p* < 0.001). (**E**) Distributions of the number of total sessions a channel responded to attempted left wrist extensions (among all channels in active footprint), by area. The median number of sessions a channel within the active footprint was observed responsive was greater among sensory than motor arrays (n_Median, M1_–n_Median, S1_ = -2 days, *p* = 0.183).

We measured longitudinal spatial stability over the 11 sessions, representing it in two ways (Fig. 3). First, we plotted frequency heat maps of channel activity across MEAs (Fig. 3A). Secondly, we estimated channel survival probability (Fig. 3B,C), defined as the chance a channel within an area or hemisphere was activated for at least *n* total sessions (1 ≤ *n* ≤ 11 for the left wrist, 1 ≤ n ≤ 8 for the right﻿), not necessarily consecutive. To calculate these probabilities, we first counted the number of channels in a location that were active for at least *n* sessions, then divided these counts by the total active channels in that location.

Contralateral hemisphere arrays (right hemisphere: Pedestal C) had concentrations of highly stable channels (active > 75% of sessions, indicated by warmer colors), which were surrounded by zones of relatively less stability (cooler colors). This pattern resembles a “center of gravity” arrangement seen in fMRI mapping literature. In the motor array, longitudinal spatial stability was highest on the right side of the array (lateral to the brain midline), and declined progressively toward the left (medial) side. In the sensory array, by contrast, the most stable channels were more dispersed, although highest stability tended to lie toward the bottom left (anterolateral) section of the array. Right ECR contractions evoked activity in center-of-mass arrangements in the contralateral (left) hemisphere, near the bottom right of the motor array and distributed throughout the sensory (Fig. S3A, Pedestal B).

Ipsilateral arrays (Fig. 3A, Pedestal B) showed only sporadic channel activity. This was largely restricted to < 25% of sessions and exhibited no clear centers of gravity. Median “lifetime” of contralateral channels was higher than ipsilateral (Fig. 3D; n_Median, Co_–n_Median, Ip_ = 4.5 sessions; *p* < 0.001, Wilcoxon signed-rank test). Altogether, longitudinal spatial stability in contralateral channels was higher than in ipsilateral, and displayed a center of gravity pattern that was absent in ipsilateral arrays. Array activity for right ECR contractions paralleled that of the left ECR: the ipsilateral (right) hemisphere lacked centers of gravity (Fig. S3A, Pedestal C), and had shorter median “lifetimes” than the contralateral (left) hemisphere (Fig. S3D, n_Median, Co_–n_Median, Ip_ = 2 sessions; *p* < 0.001).

Grouping channels by area, we found the median “lifetime” of sensory channels to be higher than of motor, both for the left ECR (Fig. 3E; n_Median, S1_–n_Median, M1_ = 2 days, *p* = 0.183; Wilcoxon signed-rank test), and the right (Fig. S3E; n_Median, S1_–n_Median, M1_ = 5 days, *p* < 0.001). Thus, sensory channels had higher longitudinal spatial stability than motor channels.

To rule out whether our maps were influenced by differences in baseline activity of our electrodes, we compared raw baseline firing rates (Fig. S4). There was a significant main effect of array implant area on baseline spiking activity (Fig. S4A; 1-way ANOVA, *p* < 0.001). In particular, baseline activity across sensory arrays was modestly higher than across motor (mean difference = 4.88 spikes/sec), likely due to their construction from a lower-impedance material (see Materials and Methods) . However, there was no relationship between baseline firing rate and channel activation during ECR contraction. Namely there were no differences between active and inactive channels in motor (Fig. S4B: Wilcoxon rank-sum test, *p* = 0.0812), sensory (Fig. S4C: *p* = 0.543), ipsilateral (Fig. S4D: *p* = 0.274) or contralateral regions (Fig. S4E: *p* = 0.103).

We excluded the Pedestal A motor array from analysis because it showed no responses to left ECR contractions (Fig. 2C). The Pedestal A sensory array, however, exhibited spatial patterning and stability consistent with our observations on the Pedestal B sensory array.

### Firing strength stability within channels decreases with passage of time between measurements, and is higher in the contralateral hemisphere

**Figure 4:**
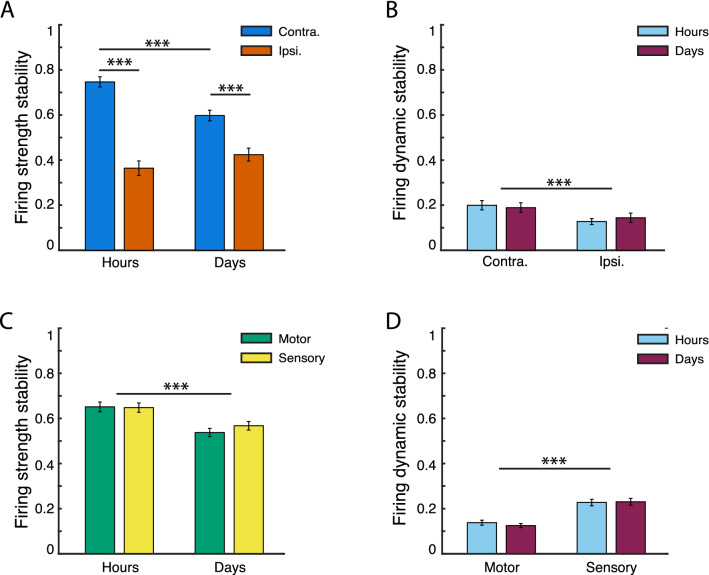
Within-channel (MUA) stabilities of left-ECR-related activity over time. X-axis denotes time scale of comparison (H: hours; D: days). Y-axis measures are normalized to the range [0, 1], higher values denoting greater stability. Bars show mean values + /- 1 SE. (*), (**), and (***) denote significance levels of 0.05, 0.01, and 0.001 respectively. (**A**) Firing strength stability significantly decreased from hours to days, and was higher for contralateral than ipsilateral channels. There was a significant hemisphere—by–timescale interaction, with a significant decrease in contralateral channel stability as the period between recordings varied from hours to days, but no corresponding significant difference in ipsilateral stability over timescale. (**B**) Firing dynamic stability (cross-correlation between z-scored PETHs) did not significantly decrease from hours to days in both hemispheres. There was a significant main effect of brain hemisphere, with contralateral channels being more stable than ipsilateral across hours and days. (**C**–**D**) Comparative stability across areas, pooled over brain hemisphere. (**C**) Firing strength stability (relative change in z-score of firing rate) by cortical area, over time. Stability significantly decreased over time from hours to days, with no significant difference between cortical areas. (**D**) Firing dynamic stability was invariant within areas from hours to days, but was significantly higher in sensory than motor channels.

Next, we compared firing strength stability (amplitude of firing rate over time) within active channels between consecutive hours (within sessions), and between consecutive sessions (across days). To measure firing strength stability for a given channel, we calculated a normalized difference of maximum PETH amplitudes for each between-hour or between-session interval being compared (see Supplementary Information for details, especially Fig. S4). Figure 4 presents the average of these stabilities across all channels, by brain hemisphere (Fig. 4A) and area (Fig. 4C).

Comparing firing strength stability between hemispheres (contralateral vs. ipsilateral hemisphere) and across timescales for the left ECR, we observed main effects of hemisphere (F(1,1048) = 176.75, *p* < 0.001) and timescale (F(1,1048) = 4.50, *p* = 0.034), with a timescale-by-hemisphere interaction (F(1,1048) = 25.03, *p* < 0.001; Fig. 4C). Stability was greater in the contralateral hemisphere than the ipsilateral for all time points of comparison (within-hours difference = 0.383, *p* < 0.0001; within-days difference = 0.192, *p* < 0.001), and decreased in the contralateral hemisphere as the interval between measurements increased from hours to days (difference = −0.322, *p* < 0.001). Firing strength stability in the right wrist extensor paralleled that of the left, with main effects of hemisphere (F(1,1210) = 52.19, *p* < 0.001) and timescale (F(1,1210) = 15.19, *p* < 0.001). Contralateral stability exceeded ipsilateral (difference = −0.147, *p* < 0.001; Fig. S6A(i)), while stability across hemispheres categorically declined as time between measurements increased (hour-vs-day difference = 0.310, *p* < 0.001; Fig. S6A(ii)). Data from both wrists revealed that as time interval between measurements increased, firing strength stability decreased, with contralateral channels being more stable overall than ipsilateral ones.

Comparing firing strength stability between area (motor vs. sensory) and timescale for left ECR, we again found a significant effect for time (F(1,1048) = 23.96, *p* < 0.001; Fig. 4A) with firing strength stability decreasing as the interval from measurements increased from hours to days (mean difference = −0.097, *p* < 0.001). For the right ECR, we similarly found a main effect of timescale (F(1,1210) = 61.15, *p* < 0.001; Fig. S6C(i)), with strength stability falling as the timescale between measurements increased from hours to days (mean difference = −0.147, *p* < 0.001). There was an additional main effect of area (F(1,1210) = 51.97, *p* < 0.001), with sensory stability being higher than motor (mean difference = −0.132).

### Firing dynamic stability within channels is greater in the contralateral hemisphere and sensory area, but preserved across timescale

We calculated dynamic stability within channels as a function of cross correlation between PETH waveforms across timescales (see Supplemental Information and Fig. S5). Figure 4 presents averages of these stabilities across all channels, assorted by brain area (Fig. 4B) and hemisphere (Fig. 4D).

When comparing firing dynamic stability for the left ECR between hemispheres, we found a significant main effect of hemisphere only (F(1,764) = 13.21, *p* < 0.001), with the contralateral being more stable than the ipsilateral (mean difference = 0.098, *p* < 0.001). For the right ECR also, the contralateral hemisphere had higher average stability (mean difference = 0.020) although there were no significant main effects of hemisphere or time.

Comparing firing dynamic stability between area and timescale for left ECR, we observed a significant main effect for area only (F(1,764) = 56.81, *p* < 0.001; Fig. 4D), sensory channels being more stable than motor (mean difference = 0.0974, *p* < 0.001). Right-ECR-related activity likewise exhibited a significant main effect for area (F(1,854) = 6.80, *p* = 0.009; Fig. S6D), with higher stability in sensory cortex than motor (difference = 0.0357, *p* = 0.012).

In sum, firing dynamic stability was higher in the contralateral hemisphere (for the left ECR), and higher for sensory channels than motor (left and right ECRs).

### Differences in channel sortability do not explain regional differences in within-channel stability

As a previous study notes^25^, neural signals defined by multiunit activity are less stable than those from single units. We therefore ran a follow-up analysis to determine whether the main effects on stability for the left ECR were due to differences in channel sortability (i.e. the main effect of brain hemisphere on strength stability, and the main effects of brain hemisphere and area on dynamic stability). First, we spike sorted our active channels (see Supplemental Information, Cluster Analysis for details). We then stratified them based on cluster volume (1, 2, or 3 + sortable units), and compared stability metrics across these subgroupings (Fig. S7).

Across arrays, sensory channels had a significantly higher number of separable units, although average yield across area was under 2 (motor: 1.171 ± 0.008 clusters per channel, sensory: 1.803 ± 0.030 clusters per channel; *t*_*8830*_ = -28.89; *p* < 0.001*,* Fig. S7A). Accordingly, the vast majority of channels (over 80%) of channels in each area (Fig. S7B(i)) and hemisphere (panel B(ii)) were separable into 1 or 2 units. Because than 1% of channels resolved into 4 or more units, we consolidated them with 3-unit channels.

Post-stratification, strength stability remained higher in the contralateral than the ipsilateral hemisphere, reaching significance for 1-unit (95% CI for contralateral = [0.632, 0.687]; ipsilateral = [0.396, 0.530]) and 3-or-more-unit groups (contralateral = [0.425, 0.609]; ipsilateral = [0.112, 0.394]; Fig. S7C). Firing dynamic stability remained higher for contralateral than ipsilateral channels, reaching significance for 1-unit (95% CI for contralateral = [0.176, 0.209]; ipsilateral = [0.122, 0.175]) and 2-unit groups (contralateral = [0.184, 0.267]; ipsilateral = [0.093, 0.162]). Dynamic stability likewise remained higher in sensory channels than motor channels, with significance among 1-unit (95% CI for sensory = [0.212, 0.270]; motor = [0.135, 0.164]) and 2-unit groups (sensory = [0.217, 0.324]; motor = [0.105, 0.178]).

Within-channel stability differences across hemisphere and area were therefore preserved after accounting for sortability.

### Ensemble stabilities are equal across timescale and area

**Figure 5:**
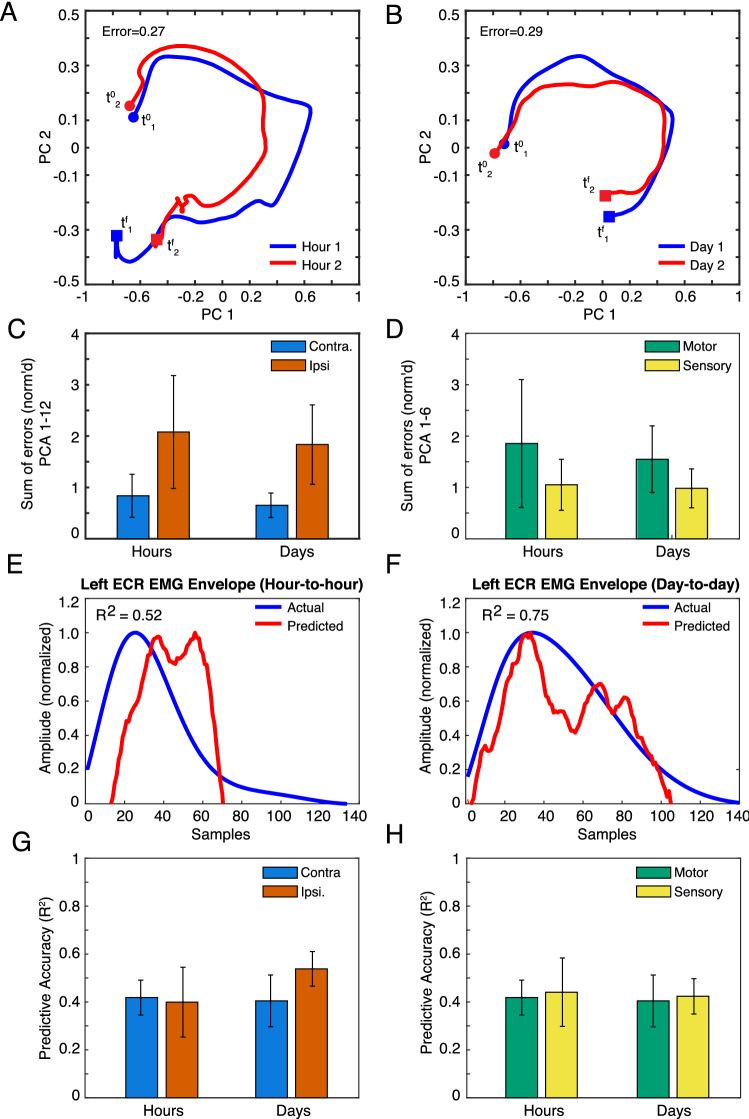
Ensemble-level (network) stabilities of left-ECR-related activity over time. (**A**–**B**) Representative principal-component (PC) trajectories of neural ensemble, visualized in the PC1-PC2 plane, during left wrist extensions across typical sets of consecutive–hour (**A**) and consecutive–day (**B**) recordings, for the right (contralateral) hemisphere (i.e. motor and sensory channels aggregated). Trajectories reflect average neural activity within EMG bursts only, with endpoints designated at the onset (filled circles, variables t^0^), and terminal points (filled boxes and variables t^f^) of the burst. Error is a normalized Euclidean distance between trajectories, with higher values indicating greater instability (see Materials and Methods). (**C**–**D**) Cumulative error between trajectory representations of ensemble activity, comprising the first six PCs, between consecutive hours, and consecutive days (Mean + /− 1 s.e). Channel ensembles are grouped by brain hemisphere (**C**) and area (**D**). (**E**–**F**) EMG of left wrist extensor (ECR: extensor carpi radialis), as measured from surface electrodes (blue) and as predicted from PCA trajectories (PCs 1–6: red) using Wiener filters trained on separate data. Data shown are for test (measurement) and training datasets recorded over consecutive hours (**E**) and days (sessions) (**F**). All envelopes are restricted to between burst onsets and terminations, and were time warped to an equal length to facilitate analysis. The x-axis shows the resulting time axis (in samples). The y-axis gives normalized envelope amplitude (see Supplementary Information). R^2^ signifies the correlation between the actual EMG and the prediction. (**G**–**H**) Correlations (R^2^) between measured (actual) and predicted EMG, arranged by brain hemisphere (**G**) and area (**H**). As above, R^2^ is based on training and test sessions spaced over consecutive hours and days (sessions).

To measure ensemble stability for the left ECR, we calculated neural trajectories across brain hemispheres and areas using principal component analysis (PCA). Visualizing over the first and second PCs (Fig. 5A,B, “PC1-PC2 plane”), we found marked similarity of trajectory shape, orientation, and amplitude between consecutive hours (A), as well as consecutive sessions (B). Expanding the analysis to the top 6 PCs, differences in latent variable trajectories did not significantly change on average, regardless of whether sessions were hours or days apart (C–D), indicating stability with time passage. Likewise, trajectory differences were unaffected by brain hemisphere (C) and area (D).

Given that such trajectory representations of ensemble activity are of wide interest for BMI decoders, we then asked how the equivalence in PC stability that we observed over time, area, and hemisphere would translate to decoding muscle activity. To examine decoder stability, we trained a Wiener filter model relating PCA trajectories to muscle EMG envelopes (see Materials and Methods, and Supplementary Information for details). For each pair of data blocks spanning consecutive hours or sessions, we performed a two-fold cross validation procedure. Namely, we trained the Wiener filter on one block, and used the fitted model to predict the time course of the EMG envelope of the left ECR on the other block. We repeated this process after switching the training and test blocks, and then averaged the goodness-of-fit measures over both cross-validation fits. Depending on whether latent variable inputs derived from motor or sensory inputs, or both, we adjusted the model to account for different input latencies (see Supplementary Information, and Fig. S9 for neural latency data to verify assumptions). Typically, there was moderate-to-high correlation between predicted and measured EMG, whether training and test sessions were hours (Fig. 5E; R^2^ = 0.52) or days apart (Fig. 5F; R^2^ = 0.75). Decoding performance was not significantly affected by the time frame of comparison, nor by area (Fig. 5G) or hemisphere (Fig. 5H). Including more PCs did not affect our conclusions (m = 9 and 12 PCs)^24^.

Ensemble-level stability analysis for right ECR contractions (Fig. S8A-H) showed main effects of hemisphere on trajectory error (F(1, 23) = 15.83, *p* = 0.006) and EMG prediction accuracy (F(1,25) = 4.75, p = 0.040) not present with the left ECR, with the contralateral hemisphere having lower between-trajectory error (mean difference = −1.55), but higher EMG decoding accuracy (mean difference = 0.180).

## Discussion

Here, we studied the characteristics and the stability of two wrist muscle representations in the sensorimotor cortex across multiple areas, time intervals and scales of resolution in the first human to have received bilateral MEA implantations. These questions are crucial, in light of practical experiences that decoders for human BMIs need to be retrained often throughout use sessions. Our main findings were: (1) Channel-level firing strength stability decreased with increased passage of time between data comparisons; (2) Within-channel stability was higher for contralateral than ipsilateral channels for both firing strength and dynamics, and higher for sensory channels than motor in firing dynamics only; (3) At the ensemble level, no stability differences were observed in manifold trajectories, either over time or by brain area.

Our results do not appear to be influenced by learning or recording quality; rather they suggest inherent properties of the brain. The task itself involved normal movements within our participant’s capacity, and as such did not show asymptotic error patterns resembling a learning effect. Thus, it is unlikely that our stability measures are influenced by neural correlates of skill acquisition^37^. Likewise, we did not find discrepancies in signal quality across brain areas or hemispheres that would account for our findings. Baseline spiking activity showed no systematic differences between active and inactive channels across area or hemisphere (Fig S4). All stability measures were based on relative firing rate changes and controlled for false positives. Spiking activity exhibited low false negative rates (see Fig. S9)^38^.

Overall, our findings suggest that the stability of muscle contraction representations in the sensorimotor cortex is heterogeneous across area and hemisphere, and that these differences can be compensated with population-wide (ensemble-level) approaches.

### Within-channel stability decreases within short timescales

The motor and sensory body map representation found here agrees with previous findings using non-invasive techniques. In motor areas, we found a concentrated area of maximal stability (Fig. 3A, array MC) akin to a “center of gravity” arrangement^39^ with a surrounding penumbra of less stable channels that disappear and reappear intermittently over sessions^40^. In sensory cortex we found that maximally stable channels were more interspersed, consistent with descriptions of S1 somatotopy having multiple dispersed “centers of gravity”^41^.

The longitudinal mapping of the left-wrist extensor suggests that the somatotopic map may be inherently unstable at the multiunit level, with minimal to no channels being active consistently in all sessions. Within-channel firing strength and dynamic stabilities declined within days, and often sooner within experimental sessions (i.e. hours). Destabilizations of within-channel unit activity within hours and days could be explained by a few factors. For example, MUA signal attributes themselves may have been inconsistent. Because firing rates derive from thresholded activity^42^, our measures are ultimately related to the action potential amplitudes, which previous studies have found often change progressively over hours and days^19,20,43^. Also, MUA stability reductions as the time between measurements extends over days could reflect neural plasticity due to our participant’s activities during the intervening times. Namely, previous work has shown that performing novel motor control tasks^24,44^ as well as consolidation during sleep after BMI use^45^ can produce detectable changes in unit-level activity.

### Contralateral MUA is more stable than ipsilateral for longitudinal spatial stability and firing strength

Sensory and motor channels were more stable in the contralateral than the ipsilateral hemisphere. While contralateral channels were active during all sessions (see e.g. Fig. 3B, left panel), the probability of observing any ipsilateral channel for more than half of sessions was zero (see e.g. Fig. 3B, right panel). Comparing the median number of sessions that a channel in each area was present, we found that the “lifetime” of a contralateral channel was higher, indicating that ipsilateral representations are inherently more transient. Within sessions, firing strength stability was greater in the contralateral hemisphere than the ipsilateral for all time points of comparison (Fig. 4A). These findings align with a previous fMRI investigation showing the contralateral hemisphere was more consistently active than the ipsilateral representation when executing an arm movement consistently over multiple days^46^.

### Sensory MUA is more stable than motor in longitudinal spatial stability and firing dynamic stability

Sensory activity was more stable than motor. Longitudinal spatial stability was higher for sensory arrays, as reflected in a shallower roll off in survival probability (Fig. 3C, left panel. Firing dynamic stability was also higher (Fig. 4B). Greater S1 stability does not appear to be related to unit sortability (Fig. S2), but rather may be an intrinsic property of region. This seems consistent with our current understanding of the relative functional roles of sensory and motor cortices: sensory processing favors stability and efficiency^47,48^, whereas motor control favors flexibility and redundancy, particularly in uncertain environments or while learning new skills^49^.

The stability discrepancy could be influenced by different material composition of our motor and sensory arrays as well (see Materials and Methods). Motor array electrodes were platinum tipped (Pt), and sensory array electrodes were sputtered—iridium—oxide—film tipped (SIROF). SIROF-tipped electrodes may also have better signal retention properties than Pt-tipped electrodes^50^, which could affect both of our stability measures.

### Ensemble-level activation is stable and overrides temporal and regional differences

At the ensemble level, we found no significant differences in stability across time (Fig. 5A–D) or between regions (Fig. 5C,D), as measured by the errors between PC trajectories.

High channel-level instability may be a natural consequence of the redundancy the nervous system affords in programming movements^23^. Toward this argument, studies in non-human primates (NHPs) show that, despite the replicability of movements such as arm reaches, neural firing patterns at the unit level may often vary quite significantly due to changes in tuning characteristics^20,22^.

Alternatively, it is possible that signal instability may result from limitations in recording hardware^19,25,50^. Latent variable analysis tempers sources of variation by representing neural activity in terms of dimensions (factors) that have optimally high explanatory ability. Despite the underlying sources of the inter-area and inter-hemispheric differences in single-channel stability that we found, PCA suggests that variations in firing rates at the ensemble-level are comparably stable regardless of implant area or hemisphere.

When we utilized these network-level representations to decode EMG from wrist extensions limited to the left ECR, we additionally found that both areas and hemispheres were equally predictive of muscle activation, and that there were no differences in accuracy across time (Fig. 5E–H). This observation is consistent with recent findings in NHPs^24^, although to our knowledge we provide the first evidence in a human.

### Sensorimotor array activity is widely distributed spatially and temporally

A distinction of our study is that we employed isolated muscle contractions, and validated their compliance with instructions using both guidance from physical therapists and EMG in real time. Our cortical activity map (Fig. 2) showed intermixed muscle representations in motor and sensory arrays, which aligns with previous characterizations. All contractions predominantly elicited activity in the array contralateral to the muscle, although we detected ipsilateral activity in both motor and sensory cortices, with ipsilateral activity in sensory cortices being somewhat more frequent (Fig. 2B).

Such bilateral activity may arise from transcallosal signaling, serving to integrate bimanual information or modify sensory response on the contralateral side^51,52^. Although synapses from callosal projections have been observed in the wrist and hand regions of Area 1 in studies of NHP histology^53,54^, our observations are possibly the first demonstration of their existence at the MUA-level in a human.

As expected, motor activity preceded sensory activity (Fig. S3). Because our participant’s injury graded B on the ASIA Impairment Scale^55^, with substantial tactile sensitivity in the hand, much of the sensory response that we observed is likely from afferent sensory feedback. Interestingly, some sensory channel activity preceded typical afferent delays (approx. 30 ms post-EMG burst) or were simultaneous with EMG burst (Fig. S2, pink shaded region). We conjecture that these early responses may be indirect evidence of efference copy signaling^9^, although further experiments are necessary to verify this.

### Implications for BMI

Practical use of BMI technology relies on developing stable decoders. Our findings suggest that decoders that weight neural activity based on the temporal dynamics of specific channels will have limited longevity. This is because the underlying neural code at the individual-multiunit level changes over short timescales, especially in the motor cortex. Although this feature may be biologically beneficial (i.e. the brain encodes motor commands in a flexible manner), it also complicates the design of neural decoders. This is because a particular combination of neural activity generated by a muscle contraction at one time expectedly leads to changes in neural activity in future iterations of the same contraction. Therefore, a better long-term strategy may be to account for a thorough description of neural activity embodied in the overall dynamics of the response across channels, as this both equalizes stability across brain regions and extends the time frame that it is maintained before declining^56^. Along this line, recent advances in decoding algorithms that consider whole-trial dynamics and activity patterns at a higher level of abstraction rather than individual electrodes^24,56^ could both provide longer lasting BMI-decoders, as well as overcome stability differences measured across brain regions.

## Materials and methods

### Study participant

As part of a study conducted under an Investigational Device Exemption (IDE) by the FDA (G170010), we recruited a volunteer participant with a C5/6 incomplete ASIA Impairment Scale (AIS) B spinal cord injury^55^ (male, 49 years old at time of surgery, 32 years post injury at time of study; right-hand dominant). The study was approved by the Johns Hopkins School of Medicine Institutional Review Board (IRB) and NIWC Pacific Human Research Protection Office (HRPO). All experiments and methods were in accordance with relevant guidelines and regulations. The participant gave informed consent to participate and to have videos and images of him used for presentation and publication.

The participant was implanted bilaterally with six cortical microelectrode arrays (NeuroPort; Blackrock Neurotech, Salt Lake City, UT): 4 in the dominant (left) hemisphere (motor array, pedestal A = MA; motor array, pedestal B = MB; sensory array, pedestal A = SA; sensory array, pedestal B = SB) and 2 in the nondominant (right) hemisphere (motor array, pedestal C = MC; sensory array, pedestal C = SC; Fig. 1A). The participant had residual control of shoulder and wrist muscles and quasi-intact sensation. All motor array electrodes were platinum tipped (Pt), and all sensory array electrodes were sputtered—iridium—oxide—film tipped (SIROF).

### Biosignal recordings

#### EMG recordings

During each block, we measured 8 channels of EMG from the targeted muscle, its contralateral homologue, adjacent muscles likely to be co-contracted, and select postural muscles (AMT-8; Bortec Biomedical, Calgary, AB, Canada). We recorded EMG and photodiode data at a 2 kHz sampling rate with Blackrock Neural Signal Processors (Blackrock Microsystems, Salt Lake City, UT), and inspected these signals online using the Central software suite (Blackrock Microsystems). We coded all experimental software and analyses in MATLAB (MathWorks, Natick, MA).

#### EMG screening and onset estimation

For each trial, we first epoched EMG signals for all recorded muscles into a window from −0.750 to 2 s relative to the metronome cue (see Fig. 1). Then, we visually screened EMG activity for co-contractions, spasms (synchronous high-amplitude global muscle activations), or missed cues (lack of EMG response from an intact target muscle). Afterward, we manually tagged EMG burst onsets and endings using a custom graphical user interface.

#### Neural recordings

We recorded multiunit activity (MUA) data by automatically thresholding 30-kHz-sampled neural data with Neuroport Neural Signal Processors (Blackrock Neurotech, Salt Lake City, UT), at a level of −3.25 dB relative to quiet sitting. Prior to offline analysis, we binned spiking times into peri-event time histograms (PETHs) at a resolution of 1 ms. Subsequently, we computed all neural activity measures on raw PETHs, only smoothing them for visualization purposes (using Gaussian kernels of width 120 ms) or for ensemble analyses (width 150 ms). We coded all experimental software and analyses in MATLAB.

### Body map experiment

We characterized the body sensorimotor map and its underlying MUA firing rate activity within the coverage of the MEA implants for the wrists (Fig. 2). To evoke muscle activity, we asked the participant to perform (or absent functional movement, to attempt) paced and repeated isolated muscle contractions to a metronome, while simultaneously recording surface EMG and MUA (Fig. 1B). We assessed each muscle in separate blocks of trials. Prior to each block, we made sure the participant was positioned to eliminate postural and extraneous EMG activity outside the targeted muscle. The participant then rehearsed the specified muscle contraction while a trained experimenter monitored EMG activity and provided verbal feedback to facilitate proper muscle isolation. After isolating the target muscle activity, we recorded EMG and MUA, while an experimenter continuously monitored EMG for task compliance and proper muscle isolation. Each block consisted of well-isolated, repeated muscle contractions paced with a metronome for approximately 2.5 min (i.e. roughly 35 trials). The metronome cue occurred every 4 s and was presented simultaneously as an auditory beep and an onscreen flash of a gray patch (of duration 0.750 s). A photodiode marked the cue onset for referencing during offline analysis. We excluded from further analysis trials where an EMG-producing muscle did not generate a recognizable signal or we observed extraneous muscle activity.

To rule out that neural activity was induced by posture or the metronome cues themselves, we repeated the experiment by having the participant sit in the same posture used during muscle contraction blocks, but passively listen and watch the metronome cues without moving.

### Sensorimotor stability experiment

To investigate stability of the sensorimotor map, we repeatedly characterized the sensorimotor activity and representation of the left and the right extensor carpi radialis longus (ECR) muscles. We focused on the ECR because it had a profuse representation among our arrays and could be reliably activated in an isolated manner without causing fatigue. We repeated the same metronome experiment described above across 11 sessions for the left wrist, and 8 sessions for the right (approximately twice per week over the course of several months; Table S1). During multiple sessions, we mapped ECR activity twice, approximately 2 h apart. Day-to-day stability compared activity from one session to the next (roughly 4 days apart). Hour-to-hour stability compared activity collected at the beginning and end of a session (roughly 2 h apart). We performed all analyses on trial-averaged PETHs from each block.

### Sensorimotor map

We constructed (1) wrist body maps (Fig. 2); and (2) stability maps of the left and right ECR to track somatotopy and individual channel activity over various timescales (Fig. 3A). For *regional body maps*, we labeled channels based on whether they were active for contractions of individual muscle groups (384 channels × 17 epochs x *m,* where *m* was number of isolated muscles per region, correcting for multiple comparisons using the False Discovery Rate, or FDR, method; see Supplemental Information^57,58^). For *stability maps*, we labeled channels according to the percentage of days that significant activity was registered (384 channels × 17 epochs × 12 days, corrected for multiple comparisons using FDR).

### Stability definitions

#### Longitudinal spatial stability

We defined longitudinal spatial stability as frequency of sessions that a wrist contraction evoked activity on each electrode, and display these results as a heat map for each array (Fig. 4A). We estimated probabilities of survival (i.e. of being active for at least n sessions of 11 for the left wrist, and of 8 for the right) by counting the number of channels per area/ hemisphere active for at least n sessions, and converting these counts to probabilities by normalizing to the total number of active channels within that area/hemisphere.

#### Within-channel stability

We characterized unit-level stability of left ECR representation in terms of firing rate and firing dynamics. Firing strength stability is the normalized difference of z-scored PETH amplitudes. Firing dynamic stability relates to correlation magnitude between aligned PETH waveforms, thus capturing more temporal features. Both metrics range from 0 to 1, higher values indicating greater stability. Computational details are available in Supplementary Information.

### Multi-channel (ensemble) stability and decoding

The goal of our multi-channel analysis was to compare the population activity across electrodes implanted in common areas or hemispheres, in terms of both their similarity and their relationship to cued muscle activations in the left wrist extensor (ECR). For averaging purposes on compliant trials only, we manually marked the EMGs for the beginning and end of the ECR contractions to use as endpoints for a common time axis across trial, and then time-warped all EMG and neural signals to this axis by linear interpolation (150 points). Afterward, we averaged time-warped data by block.

In each block, we grouped neural channels into ensembles by brain area (motor/sensory), or hemisphere (contralateral/ ipsilateral), and analyzed them using principal component analysis (PCA). To assure that resulting PCs for each ensemble were uniform across all hour-to-hour and all day-to-day (session-to-session) blocks, we first concatenated all blocks of ensemble-specific data for each time scale (hours or days), and then decomposed each ensemble-specific concatenation with a single PCA.

To measure stability of the mapping between PC structure and EMG, we used a Wiener-filter approach, detailed in Supplementary Information.

### Statistical analyses

We compared differences in longitudinal spatial stability by measuring the survival rates across time for channels of each area (motor, sensory) and hemisphere (contralateral, ipsilateral). Differences in the distribution of the number of sessions the channels between areas or hemispheres were assessed using nonparametric unpaired two-sample tests (Wilcoxon signed-rank test).

To compare within-channel firing rate and firing dynamic stability, we performed 2-way ANOVAs (1) with factors of brain area (motor, sensory) and timescale (hours, days), and (2) with factors of laterality (contralateral, ipsilateral) and timescale (hours, days). We also relate to upper bounds by comparing mean values with 95% CIs of bounds.

We report all data as means ± 1 s.e. Effects were considered significant if *p* ≤ 0.05. All post-hoc analyses were done using two-tailed Tukey’s tests of Honestly Significant Difference (HSD).

## Supplementary Information

Supplementary Information.

## Data Availability

The data used and analyzed during the current study is available from the corresponding author on reasonable request.
