# Supplementary material for: Characteristics and stability of sensorimotor activity driven by isolated-muscle group activation in a human with tetraplegia
Source: Sci Rep. 2022 Jun 20;12:10353. doi: 10.1038/s41598-022-13436-2 (PMC9209428; doi:10.1038/s41598-022-13436-2)
Supplement: Supplementary file 1 — Supplementary Information. [file 41598_2022_13436_MOESM1_ESM.docx]

Supplementary Information for

"Characteristics and stability of sensorimotor activity driven by isolated-muscle group activation in a human with tetraplegia"

Robert W. Nickl^[[1]](#footnote-1)^, Manuel A. Anaya^1^, Tessy M. Thomas^2^, Matthe­­w S. Fifer^5^, Daniel N. Candrea^2^, David P McMullen^6^, Margaret C. Thompson^5^, Luke E. Osborn^5^, William S. Anderson^4,^ Brock A. Wester^5^, Francesco V. Tenore^5^, Nathan E. Crone^3^, Gabriela L. Cantarero^1^, and Pablo A. Celnik^1,2,*^

SUPPLEMENTAL METHODS

Map and stability analyses were primarily done on multiunit activity (MUA). During experiments, MUA signals were thresholded online (threshold level = -3.25dB relative to baseline activity measured at rest) and saved as a sequence of spike times (sampled at 30,000 Hz). Offline, we calculated spike times to firing rates by binning them at a frequency of 1 kHz to generate peristimulus time histograms (PETHs).

Most analyses were performed on firing rates represented by PETHs. However, as a side analysis we ran a spike sorting algorithm to study the relationship between stability and unit separability in our data (see section on *Cluster Analysis* section below).

**Active Channel Detection**

To determine body maps for the wrist muscles as in Figures 2C, 3A, and S3A, we labeled the recording channels on our participant’s arrays as active or inactive, based on the significance of their measured MUA. We determined which channels were active during contractions of wrist extensor and flexor muscles of each arm using a sliding window method (Fig. 1C).

For a given channel, *baseline activity* for each trial was calculated as the average firing rate (PETH amplitude) over a fixed window spanning -0.500s to -0.250s (relative to the EMG burst onset for that trial). *Response activity* for each trial was calculated at increments of 0.125s by sliding a window (length=0.250s, step width=0.125s) over the PETH from -0.250s to 2s and averaging the PETH amplitude within these windows. We detected MUA activations by comparing the average response firing rate for each window (17 total) to the mean baseline firing rate using separate two-sided statistical tests per channel. A paired t-test was used for channels with a normal distribution, and a two-sided Wilcoxson signed-rank test for non-normal distributions. We ascertained normality using Lilliefors tests. After correcting for multiple comparisons across channels and muscles using the false discovery rate (FDR) method at q = 0.05^[1,2]^, we labeled as active any channels having responses significantly different from baseline for at least 1 window. Channels that showed clear signs of muscle artifact were excluded from further analysis.

**Channel Baseline Measurements**

To quantify raw activity over our electrodes, we calculated baseline channel firing rates for all channels across all blocks of our study (Fig. S4). In all cases, baselines derived from a window -0.5 to -0.25 s relative to the EMG burst onset. All firing rates correspond to multiunit activity.

We compared raw activity at two levels: (1) between arrays (Fig. S4A, arrays ordered by increasing median firing rate) and (2) between active and inactive channels assorted by brain area (Fig. S4B-C) and hemisphere (Fig. S4D-E).

**Within-Channel Stability Calculations**

All within-channel stability calculations (Figs. 4 and S6) were done on PETHs for channels showing significant responses only.

*Firing rate stability:* Firing rate stability refers to the consistency of PETH amplitudes (normalized to baseline) across time, defined per channel as:

$$1- \frac{\left| z_{t1}-z_{t2} \right|}{\left| z_{t1} \right|+\left| z_{t1} \right|}$$

where z_t1_ and z_t2_ refer to average PETH amplitudes in a window of -0.25 s to 0.25 s relative to the EMG burst, z-scored relative to baseline at measurement time points indexed by t1 and t2 (which may be separated by days or hours). We computed firing rate stability for each channel that was active during at least one endpoint of each time comparison (hour-to-hour, day-to-day).

*Firing dynamic stability:* Firing dynamic stability measures the similarity between the shape of the PETH waveforms at the two measurement times t1 and t2. It is defined per channel as the absolute value of the zero-lag cross-correlation between the z-scored PETHs within the aforementioned window about the EMG-burst. We calculated firing dynamic stability for each channel that was active during at least one endpoint of each time comparison (hour-to-hour, day-to-day).

Figure S9 shows example data that highlights changes in both within-channel stability types across consecutive sessions. We signify firing rate stability by $\Delta z$, and dynamic stability by $\rho$ .

**Cluster Analysis**

Because previous studies have related channel stability to multiunit separability, i.e. single units may be more stable than multi-units^27^, we evaluated whether the number of separable single units influenced our stability metrics (Fig. S7). To do this, we sorted all channels into 1-cluster (i.e. single unit), 2-cluster, (i.e. multiunit) or 3-or-more -cluster (multiunit) groups using the unsupervised method wave_clus^[3-4]^. The average number of clusters per array tended to between 1 and 2 (Fig. S7A), with the vast majority of channels exhibiting at most 2 clusters (Fig. S7B). After grouping channels by cluster quantity (1, 2, or 3+), we bootstrapped distributions of strength stability by brain hemisphere (Fig. S7C) and dynamic stability (Fig. S7D) by area and hemisphere. In each subpanel, markers denote the stability scores (firing strength or firing dynamic) of individual bootstrap experiments, and bars represent 95% confidence intervals of these scores.

**Decoder Calculations**

To measure the stability with which neural ensemble activity (expressed in PCs) encoded muscle activity during left wrist extensions, we applied a decoding approach similar to Gallego et al.^[5]^ Since we were interested in comparative stabilities between brain area and hemisphere, we trained decoders based on PETHs across 4 subsets (ensembles) of neural microelectrode data: all motor channels, sensory channels, contralateral channels (right hemisphere), and ipsilateral (left) channels. Before applying PCA, we culled out artifactual channels, and applied a square-root transform to all remaining PETHs. Outputs in all cases were the EMG envelopes from 6 muscles, comprising the wrist extensor, wrist flexor, and thumb adductor of each arm. Envelopes were filtered at 5 Hz, and for the purposes of model fitting, were normalized to the maximum amplitude of the left ECR envelope. The latter step served to mitigate variations in skin contact for electrodes across blocks.

The decoder model used was a Wiener filter, of the following form:

$$y\left[ n \right]=\sum_{m=M1}^{M2} \left. A_{m}x[n-m \right]$$

Here, x[n] is the filter input, defined by the first 6 principal components across a channel ensemble at time point n; y is the filter output, defined as the vector of EMG envelopes over the above listed muscles at time n; and A_m_ is the vector of mixing coefficients relating neural input to muscular output. Index m denotes the number of time instants (or “taps”) factored into the model before (m > 0), or after (m < 0) the instant of EMG measurement. We downsampled all data at 100 Hz, and considered inputs comprising (1) motor array channels only, (2) sensory array channels only, and (3) both array types. To model the EMG output at time n, we included the previous 100 ms (10 timesteps) of neural data for motor-input models (M1= 0, M2 = 10), the following 100 ms (10 timesteps) for sensory-input models (M1 = -10, M2 = 0), and a symmetric window of 100 ms (M1 = -5, M2 = 5) for mixed-input models. These values comport with those used in Gallego et al.^[25]^, and agree with neural latency estimates from our data (Fig S9).

For each pair of consecutive data blocks (spaced by hours or by days/sessions), we designated one block as a training set, and the other as a test set. We estimated the Wiener filter using the PCs on the first data set of the temporal pair, and cross validated this filter against the neural activity and EMG of the test data set. Stability was measured as the correlation between actual and predicted EMG of the left ECR with R^2^ (see Fig 5E-F for the left ECR; Fig. S8E-F for the right). We then interchanged the training and test sets and repeated this process. Both R^2^ values were then averaged to yield an overall goodness of fit for the consecutive-timepoint comparison, and the overall process was repeated across all pairs of consecutive times. This process, when carried out for all possible neural data ensembles (motor/sensory or contralateral/ipsilateral, yielded the distribution of R^2^ values presented in Fig 5G-H for the left ECR (Fig. S8G-H for the right).

**Multiunit Latency**

We estimated latency distributions for MUA evoked by ECR contractions, in order to verify assumptions of our Wiener-filter decoding model (shown in Fig S9). First, for each active channel, we averaged the EMG-referenced PETHs over trials. Then we estimated latencies based from times when mean PETHs crossed a threshold (the temporal mean of the baseline window plus or minus a multiple of its temporal standard deviation) and remained above or below for at least 0.050 s consecutively. For left ECR, we set this multiple to 3.5 times baseline s.d. This multiple approximately equaled ­the Bonferroni-corrected threshold labeling a channel active (0.05 significance level), and is comparable to other published latency thresholds^[6-10]^. In final analysis, we rejected as outliers any data outside the median + 1.5 times the interquartile ratio (corresponding to average retentions of 88.4% of motor and 82.8% of sensory values).

Latencies for the left ECR were approximately Gaussian distributed for both motor and sensory activity and centered relative to EMG burst onset (Fig S3). Average motor latencies preceded sensory latencies (mean motor latency = x_M_ = 0.0017 +/- 0.0037 s relative to EMG onset; mean sensory latency x_S_ = 0.069 +/- 0.0022 s relative to EMG onset). Moreover, a subset of sensory latencies occurred prior to 30 ms after the initial EMG burst (denoted by inclusion in the pink shaded region of Fig S3), preceding what we expected for a normal cortico-muscular delay.

**False Negatives Calculation**

As a quality assessment for our spiking data, we applied the “false negatives from threshold detection” presented in Hill et al.^[11]^. to each session of our data (Fig S10). This measure estimates the percentage of biological spikes discarded on each channel (false negatives) by our choice of detection threshold (-3.25 dB relative to channel activity at rest).

For each channel in our array set, we compiled the distribution of amplitudes for all waveforms that passed our default threshold of -3.5 dB (relative to the average firing rate at rest). Generally, the resulting distribution assumed the form of a truncated Gaussian^[11]^. We then fit a Gaussian to this truncated distribution using the MATLAB optimization toolbox (MATLAB nlinfit), using initial parameter values of the sample mean and standard deviation estimated from the data. The area under the fitted Gaussian extending above the threshold was defined as the false negative rate for that channel. We then cycled through the remaining channels, calculated their respective false negative rates, and computed their distributions (mean + 95% confidence interval.

To compare signal quality across areas and hemispheres within sessions, we repeated this analysis for the subsets of channels corresponding to each area (Panel A) and hemisphere (Panel B).

REFERENCES

1. Benjamini, Y. & Hochberg, Y. Controlling the false discovery rate: A practical and powerful approach to multiple testing. *J. Roy.Stat. Soc. B* 57(1), 289-300 (1995).
2. Groppe, D. M., Urbach, T. P. & Kutas, M. Mass univariate analysis of event-related brain potentials/fields I: A critical tutorial review. *Psychophysiology*, 48(12): 1711-1725 (2011).
3. Quiroga, R. Q., Nadasdy, Z. & Ben-Shaul, Y. Unsupervised spike detection and sorting with wavelets and superparamagnetic clustering. *Neural Comput.* 16(8), 1661-1687 (2004).
4. Chaure, F. J., Rey, H. G., Quian Quiroga, R. A novel and fully automatic spike-sorting implementation with variable number of features. *J. Neurophysiol.* 120(4), 1859-1871 (2018).
5. Gallego, J. A., Perich, M. G., Chowdhury, R. H., Solla, S. A. & Miller, L. E. Long-term stability of cortical population dynamics underlying consistent behavior. *Nat Neurosci* *23*(2), 260-270 (2020).
6. Maunsell, J. H. & Gibson, J. R. Visual response latencies in striate cortex of the macaque monkey. *J. Neurophysiol.* 68(4), pp.1332-1344 (1992).
7. Churchward, P. R., Butler, E. G., Finkelstein, D. I., Aumann, T. D., Sudbury, A. & Horne, M. K. A comparison of methods used to detect changes in neuronal discharge patterns. *J. Neurosci. Methods* 76(2), 203-210 (1997).
8. Eifuku, S., De Souza, W. C., Tamura, R., Nishijo, H. & Ono, T. Neuronal correlates of face identification in the monkey anterior temporal cortical areas.  *J. Neurophysiol.* 91(1), 358-371 (2004).
9. Sugase-Miyamoto, Y. & Richmond, B. J. Neuronal signals in the monkey basolateral amygdala during reward schedules. *J. Neurosci.* 25(48), 11071-11083 (2005).
10. Levakova, M., Tamborrino, M., Ditlevsen, S. & Lansky, P. A review of the methods for neuronal response latency estimation. *BioSystems* 136, 23-34 (2015).
11. Hill, D. N., Mehta, S. B. & Kleinfeld, D. Quality metrics to accompany spike sorting of extracellular signals. *J. Neurosci.* 31(24), 8699-8705 (2011).

SUPPLEMENTAL TABLE

**Table S1. Schedule of sessions for stability experiment**

Table includes session timing relative to the date of MEA implantation (1/17/2019), calendar date, and whether or not two blocks were collected to make hour-to-hour stability comparisons. We note that an additional session was run (Day “409”) but excluded from our final analysis because of an abnormally low active channel yield (<10 active channels).

A. L Wrist

| # Days post implantation | Date | Hour-to-hour comparison? |
| --- | --- | --- |
| 354 | 1/6/2020 | Yes |
| 365 | 1/17/2020 | No |
| 383 | 2/4/2020 | Yes |
| 389 | 2/10/2020 | No |
| 393 | 2/14/2020 | Yes |
| 403 | 2/24/2020 | Yes |
| 537 | 7/7/2020 | Yes |
| 539 | 7/9/2020 | Yes |
| 546 | 7/16/2020 | Yes |
| 547 | 7/17/2020 | Yes |
| 550 | 7/20/2020 | Yes |

B. Right Wrist

| # Days post implantation | Date | Hour-to-hour comparison? |
| --- | --- | --- |
| 354 | 1/6/2020 | No |
| 365 | 1/17/2020 | No |
| 383 | 2/4/2020 | Yes |
| 386 | 2/7/2020 | No |
| 393 | 2/14/2020 | Yes |
| 403 | 2/24/2020 | Yes |
| 537 | 7/7/2020 | Yes |
| 539 | 7/9/2020 | Yes |

SUPPLEMENTAL FIGURES

**Figure S1: Task performance on individual days, expressed relative to the date of microelectrode implantation (see Table S1).**

(A) Error trials for each experiment session, defined by the number of non-compliant muscle contraction trials (left extensor carpi radialis: ECR). Data shown corresponds to total errors (black), and is further broken down into error classes: misses, or non-responsive trials (blue); co-contractions, or extraneous muscle activations (red); and unclean contractions, namely when a single, intentional response to the cue could not be clearly established (green).

(B) Panel A, in terms of the percentage of total trials per day.

**Figure S2: Active channel yield on individual session days for wrist extensor contractions (ECR: extensor carpi radialis) on each side of the body. All days are expressed relative to the implantation.**

(A) Total number of active channels detected per day for left ECR contractions by area (yellow: motor; green: sensory)

(B) Number of active channels per day for left ECR contractions, by hemisphere (contra/right hemisphere: blue; ipsi/left hemisphere: red). A significant correlation existed between number of contralateral channels and time (Kendall’s Tau test, significance level of 0.05). $\tau$: Tau correlation coefficient, ranging from 0 (no correlation) to 1 (perfect correlation).

(C) Total number of active channels detected per day for right ECR contractions, by area (yellow: motor; green: sensory).

(D) Number of active channels for right ECR contractions, by hemisphere (contra/left hemisphere: blue; ipsi/right hemisphere: red).

­
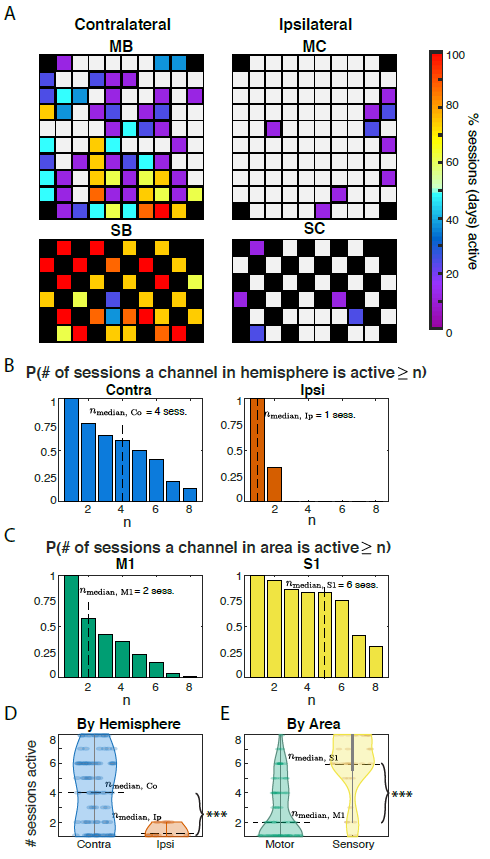


**Figure S3: Spatial patterning and longitudinal stability activity from contractions of right wrist extensor (ECR: extensor carpi radialis).**

Panels are organized as in Fig. 3 of the main text.

(A) Frequency of activity across sessions for each channel distributed over arrays B (here, contralateral hemisphere) and C (ipsilateral). The top row corresponds to the motor arrays, and the bottom to sensory. Color code denotes the percentage of sessions (of 8) that a given channel was active, with higher percentages corresponding to greater longitudinal stability.

(B) Probabilities that any active channel on Pedestals B and C responded for more than n sessions, within contralateral (left panel) and ipsilateral (right) hemispheres. Motor and sensory areas are pooled. Dashed lines mark the median number of sessions responsive among channels within the active footprint of each hemisphere.

(C) Probabilities that any active channel on Pedestals B and C responds for more than n sessions, measured for motor (left panel) and sensory (right) areas (hemispheres pooled). Dashed lines mark the median number of sessions that a given channel in the active footprint of each area responds.

(D) Distributions of the number of total sessions a channel responded to attempted right wrist extensions, by hemisphere. The median number of sessions a channel was observed responsive was greater within the contralateral than the ipsilateral hemisphere (n_Median, Co_ – n_Median, Ip_ = 2 sessions; p < 0.001)

(E) Distributions of the number of total sessions a channel responded to attempted left wrist extensions (among all channels in active footprint), by area. The median number of sessions a channel within the active footprint was observed responsive was greater among sensory than motor arrays (n_Median, M1_ – n_Median, S1_ = 5 days, p < 0.001)

**Figure S4: Baseline firing rates across recording arrays (left wrist extension blocks). Asterisks denote significance levels (*: p < 0.05; **: p < 0.01; ***, p < 0.001). Box plots are centered at the median firing rate across all sessions. All channels are unsorted.**

(A) Baseline activity over individual arrays, in order of increasing median per-channel firing rate. Baseline activity on sensory arrays was significantly higher than on motor (p = 2.02 x 10^-6^; median difference = 4.88 spikes / sec). Among motor areas, baseline activity significantly differed for each array as annotated.

(B) Baseline activity for active and inactive channels, within motor arrays (see Supplemental Methods: *Active Channel Detection*). No significant difference was detected between active and inactive channels (Wilcoxon rank-sum test, p = 0.0812; median diff = 3.58 spikes / s).

(C) Baseline activity for active and inactive channels, within sensory arrays (see Supplemental Methods: *Active Channel Detection*). No significant group difference was found (p=0.274, median difference between active and inactive channels = 2.44 spikes / s).

(D) Baseline activity for active and inactive channels, within arrays ipsilateral (left hemisphere) to the wrist extensor. No significant group differences were found

(p=0.274, median difference = 2.44 spikes / s)

(E) Baseline activity for active and inactive channels, within arrays

contralateral (right hemisphere) to the wrist extensor. No significant group differences were found (p = 0.103, median difference between active and inactive channels = 0.3 spikes / s)

.


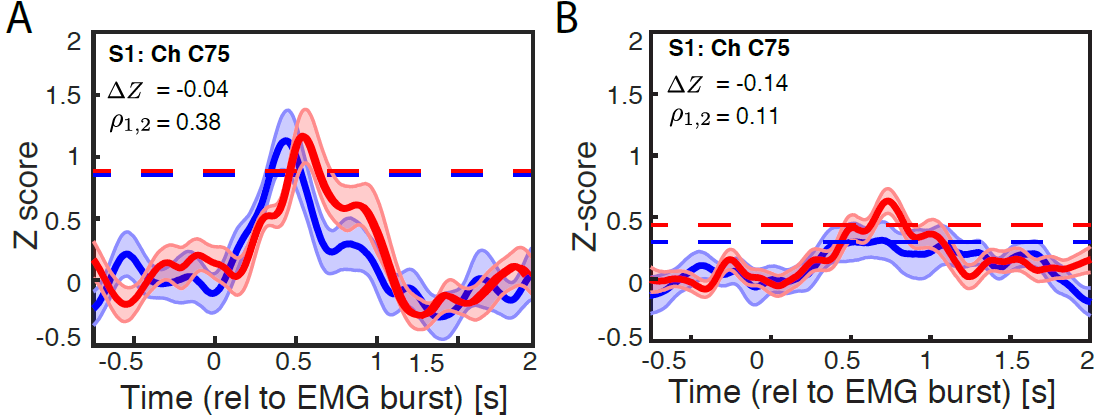


**Figure S5: Declining firing--rate and firing--dynamic stability over time for left ECR-related activity, on a representative channel (Pedestal C sensory array, Channel 75).**

(A-B) Red and blue curves denote z-scored PETHs from each endpoint of the time comparison (means +/- 1 s.e. from bootstrapping). Firing strength stability is reported as a change in average z-scored PETH over an interval [-0.25, 0.25], and is represented numerically with the variable $\Delta Z$, and graphically for each time point as a horizontal dashed line. Firing dynamic stability is given as magnitude of cross correlation (zero-lag) between waveforms in the same interval, and denoted numerically with the variable $\rho_{1,2}$.

A) Representative firing rate across two measurements on the time scale of minutes.

B) Representative firing rate across two measurements on the time scale of days. Note The overall increase in $\Delta Z$, and decrease in $\rho_{1,2}$ that are shown in panel B reflect that these waveforms become less similar across time points, and thus the within-channel firing patterns destabilize.

**Figure S6: Within-channel (MUA) stabilities of right-ECR-related activity over time.** Panel organization follows Fig 4 in the main text.

(A) Firing strength stability significantly decreased as time between recordings increased from hours to days. Strength stability showed main effects for both timescale (F(1,1210)=15.19, p<0.001; Subpanel i), and hemisphere (F(1,1210)=52.19, p<0.001; Subpanel ii). There was a significant laterality—by--timescale interaction, with a significant decrease in contralateral channel stability as the period between recordings varied from hours to days, but no corresponding significant difference in ipsilateral stability over timescale.

(B) Firing dynamic stability (cross-correlation between z-scored PETHs) did not significantly decrease from hours to days in both hemispheres. There was a significant main effect of brain hemisphere, with contralateral channels being more stable than ipsilateral across hours and days.

(C-D) Comparative stability across areas, pooled over brain hemisphere.

(C) Firing strength stability (relative change in z-score of firing rate) by cortical area, over time. Main effects on stability of the time between measurements (Subpanel i), and of brain area (Subpanel ii) were found. Stability significantly decreased as the time between measurements increased from hours to days(Subpanel i), and was greater in sensory than motor cortex (Subpanel ii).

(D) Firing dynamic stability was invariant within areas from hours to days, but was significantly higher in sensory than motor channels.

**Figure S7: Influence of channel sortability on within-channel stability.**

Annotations denote significance levels of 0.05 (*) and 0.01 (**).

(A) Number of separable units for each brain region over pedestals, as determined by wave_clus method. Data is shown for aggregate (left), and individual pedestals. Error bars are +/- 1 s.e.

(B) Percentages of channels sortable into 1, 2, or 3-or-more clusters, classified by brain area (Subpanel i) and hemisphere (Subpanel ii). Channels sortable into 4 or more clusters were included with 3-cluster channels because they accounted for fewer than 1% of all channels.

(C) Bootstrapped distributions of firing strength stability over contralateral and ipsilateral hemisphere channels, grouped by cluster yield as in Panel B. Higher values denote higher stability. Within all cluster groups, contralateral channel stability was higher than ipsilateral, reaching significance for 1-unit (95% CI for contralateral=[0.632, 0.687]; ipsilateral=[0.396, 0.530]) and 3-or-more-unit groups (contralateral =[0.425, 0.609]; ipsilateral=[0.112, 0.394]).

(D) Bootstrapped distributions of firing dynamic stability. Higher values denote higher stability.­­­­

(D(i)) Dynamic stability across hemispheres, grouped by cluster yield. Within all cluster groups, contralateral channel stability was higher than ipsilateral, reaching significance for 1-unit (95% CI for contralateral=[0.176, 0.209]; ipsilateral=[0.122, 0.175]) and 2-unit groups (contralateral=[0.184, 0.267]; ipsilateral=[0.093, 0.162]).

(D(ii)) Dynamic stability across sensorimotor areas. Within all cluster groups, sensory channel stability was higher than motor, reaching significance for 1-unit (95% CI for sensory=[0.212, 0.270]; motor=[0.135, 0.164]) and 2-unit groups (sensory=[0.217, 0.324]; motor=[0.105, 0.178]).

**Figure S8: Ensemble-level (network) stabilities of right-ECR-related activity over time.** Panel organization follows Fig 4 in the main text.

(A–B) Representative principal-component (PC) trajectories of neural ensemble, visualized in the PC1-PC2 plane, during right wrist extensions across typical sets of consecutive--hour (A) and consecutive--day (B) recordings, for the left (contralateral) hemisphere (i.e. motor and sensory channels aggregated). Trajectories reflect average neural activity within EMG bursts only. Filled circles (variables t^0^) denote burst onset, and filled boxes (variables t^f^) denote burst terminations. Error is a normalized Euclidean distance between trajectories, with higher values indicating greater instability (see *Materials and Methods*).

(C–D) Cumulative error between trajectory representations of ensemble activity, comprising the first six PCs, between consecutive hours, and consecutive days (Mean +/- 1 s.e). Channel ensembles are grouped by brain hemisphere (C) and area (D). There was a significant main effect of hemisphere, with contralateral representations having lower error (discrepancy) than ipsilateral as the time between measurements increased (F(1, 23)=15.83, p=0.006; mean difference=-1.55)

(E–F) EMG of right ECR, as measured from surface electrodes (blue) and as predicted from PCA trajectories (PCs 1-6: red). Data shown is for test (measurement) and training datasets recorded over consecutive hours (E) and days (sessions) (F). R^2^ signifies the correlation between the actual EMG and the prediction.

(G–H) Correlations (R^2^) between measured (actual) and predicted EMG, arranged by brain hemisphere (G) and area (H). As above, R^2^ is based on training and test sessions spaced over consecutive hours and days (sessions). There was a significant main effect of hemisphere only (F(1,25)=4.75, p=0.040), with contralateral-hemisphere-based EMG predictions having higher agreement with measured EMG than ipsilateral-hemisphere based (mean difference=0.180).

**Figure S9: Neural latency distributions on motor (top row) and sensory (bottom) channels responding to left ECR contractions.**

The distribution of latencies in the motor (top panel) and sensory arrays (bottom) are shown, relative to EMG burst onsets (t = 0). Vertical dashed lines labeled $\bar{x_{M}}$ and $\bar{x_{S}}$ are mean motor and sensory latencies, respectively. Pink regions highlight potential efference activity at latencies below 30 ms, Virtually all counts are from the pedestals contralateral to the side of the body of the muscle. Latencies outside of the range of the median +/- 1.5 times the interquartile range were excluded, per a conventional definition of outliers for non-parametric distributions. This amounted to retentions of 88.4% of motor and 82.8% of sensory latency values.


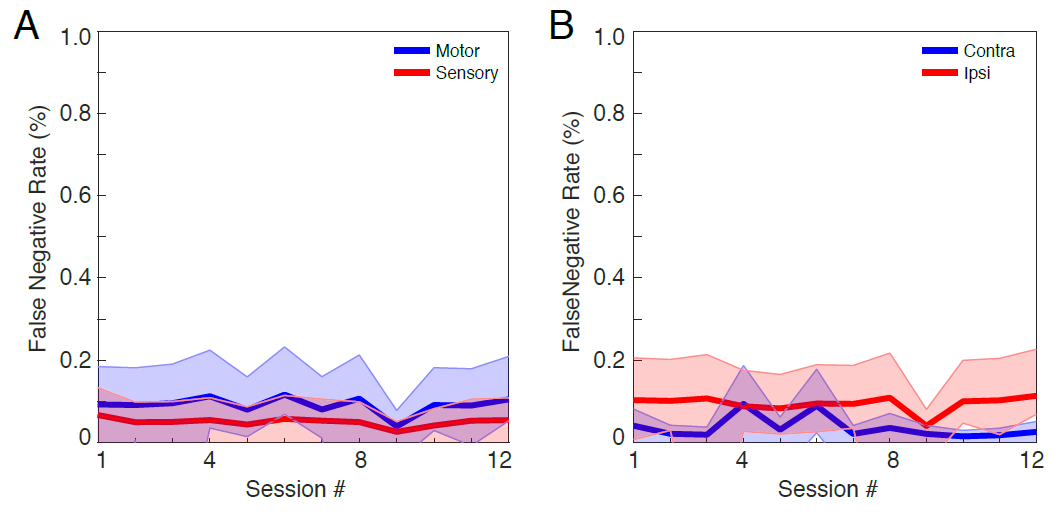


**Figure S10: False negative rates (in percentages) across all channels for each session (left wrist extension blocks), as defined in Hill et al. (2011)^62^.**

Comparisons are between: (A) Areas (blue: motor, red: sensory) and (B) hemispheres (blue: contralateral; red: ipsilateral). Solid lines denote averages, and shaded regions 95% are confidence intervals, across channels.

1. Department of Physical Medicine and Rehabilitation, Johns Hopkins School of Medicine, Baltimore, MD, USA

   2. Department of Biomedical Engineering, Johns Hopkins School of Medicine, Baltimore, MD, USA

   3. Department of Neurology, Johns Hopkins School of Medicine, Baltimore, MD, USA

   4. Department of Neurosurgery, Johns Hopkins School of Medicine, Baltimore, MD, USA

   5. Research and Exploratory Development Department, Johns Hopkins Applied Physics Laboratory, Laurel, MD, USA

   6. National Institute of Mental Health, National Institutes of Health, Bethesda, MD

   * Corresponding author: Pablo A Celnik ([pcelnik@jhmi.edu](mailto:pcelnik@jhmi.edu)) [↑](#footnote-ref-1)
